# Catechol Groups Enable Reactive Oxygen Species Scavenging-Mediated Suppression of PKD-NFkappaB-IL-8 Signaling Pathway by Chlorogenic and Caffeic Acids in Human Intestinal Cells

**DOI:** 10.3390/nu9020165

**Published:** 2017-02-20

**Authors:** Hee Soon Shin, Hideo Satsu, Min-Jung Bae, Mamoru Totsuka, Makoto Shimizu

**Affiliations:** 1 Department of Applied Biological Chemistry, Graduate School of Agricultural and Life Sciences, The University of Tokyo, Tokyo 113-8657, Japan; hsshin@kfri.re.kr (H.S.S.); japanpaper04@gmail.com (M.-J.B.); atotuka@mail.ecc.u-tokyo.ac.jp (M.T.); ms205346@nodai.ac.jp (M.S.); 2 Division of Nutrition and Metabolism Research, Korea Food Research Institute, Seongnam-si 13539, Korea; 3 Department of Food Biotechnology, University of Science and Technology (UST), Daejeon 34113, Korea; 4 Department of Biotechnology, Faculty of Engineering, Maebashi Institute of Technology, Gunma 371-0816, Japan; 5 Institutes of Entrepreneurial BioConvergence, Seoul National University, Seoul 08826, Korea; 6 Department of Nutritional Science, Tokyo University of Agriculture, Tokyo 156-8502, Japan

**Keywords:** chlorogenic acid, caffeic acid, interleukine-8, nuclear factor κB, protein kinase D, reactive oxygen species, catechol group

## Abstract

Chlorogenic acid (CHA) and caffeic acid (CA) are phenolic compounds found in coffee, which inhibit oxidative stress-induced interleukin (IL)-8 production in intestinal epithelial cells, thereby suppressing serious cellular injury and inflammatory intestinal diseases. Therefore, we investigated the anti-inflammatory mechanism of CHA and CA, both of which inhibited hydrogen peroxide (H_2_O_2_)-induced *IL-8* transcriptional activity. They also significantly suppressed nuclear factor kappa-light-chain-enhancer of activated B cells (*NF-κB*) transcriptional activity, nuclear translocation of the p65 subunit, and phosphorylation of IκB kinase (IKK). Additionally, upstream of IKK, protein kinase D (PKD) was also suppressed. Finally, we found that they scavenged H_2_O_2_-induced reactive oxygen species (ROS) and the functional moiety responsible for the anti-inflammatory effects of CHA and CA was the catechol group. Therefore, we conclude that the presence of catechol groups in CHA and CA allows scavenging of intracellular ROS, thereby inhibiting H_2_O_2_-induced IL-8 production via suppression of PKD-NF-κB signaling in human intestinal epithelial cells.

## 1. Introduction

Chlorogenic acid (CHA) is one of the most widely consumed polyphenols because it occurs abundantly in numerous foods, especially coffee. CHA has antioxidative [1], anti-inflammatory [2], anti-obesity [3], anti-diabetic [4], and anti-cancer activities [5] and owing to its biological effects, CHA has been used in various functional foods and as supplemental agents. Caffeic acid (CA) is one of the CHA metabolites hydrolyzed by esterase and gut microflora in the gastrointestinal tract. However, CA is a phenolic acid that also occurs naturally in numerous agricultural products such as fruits, vegetables, wine, olive oil, and coffee. Similar to CHA, CA has been reported to have antioxidant, anti-tumor, and anti-inflammatory activities [6,7,8]. In a previous study, we reported that oxidative stress-induced interleukin-8 (IL-8) secretion and mRNA expression were markedly inhibited by CHA. Furthermore, its metabolite CA, suppressed IL-8 production but not that of quinic acid (another metabolite of CHA) in Caco-2 cells [9]. Recently, it was reported that CHA and CA reduced the mRNA expression of macrophage inflammatory protein 2 (MIP-2, a mouse homologue of *IL-8*) in a dextran sulfate sodium-induced colitis model [10]. However, the inhibitory mechanism of CHA and CA on *IL-8* production has not yet been fully elucidated. 

Oxidative stress can trigger toxicity, damage, and inflammation in cells. In general, reactive oxygen species (ROS) including singlet oxygen (^1^O_2_), hydrogen peroxide (H_2_O_2_), superoxide (O_2_^–^), and hydroxyl radical (HO•) develop antioxidant defenses by enhancing the expression of superoxide dismutase, catalase, glutathione peroxidase, and peroxiredoxins, which maintain the redox balance [11]. However, when cellular ROS production overwhelms the antioxidant capacity, the ROS causes oxidative stress-induced serious cell damage via production of toxic chemical compounds and cytokines/chemokines, that results in the pathogenesis of several diseases. For example, oxidative stress promotes the production of inflammatory cytokines in intestinal epithelial cells. Among the inflammatory cytokines, IL-8 in particular, plays a key role in the pathogenesis of inflammatory bowel diseases (IBDs), which include Crohn’s disease and ulcerative colitis [12]. Therefore, the inhibition of IL-8 as a therapeutic target is essential in controlling inflammatory diseases like IBDs. Nevertheless, the mechanism underlying the regulation of IL-8 has not yet been fully elucidated. 

**Figure 1:**
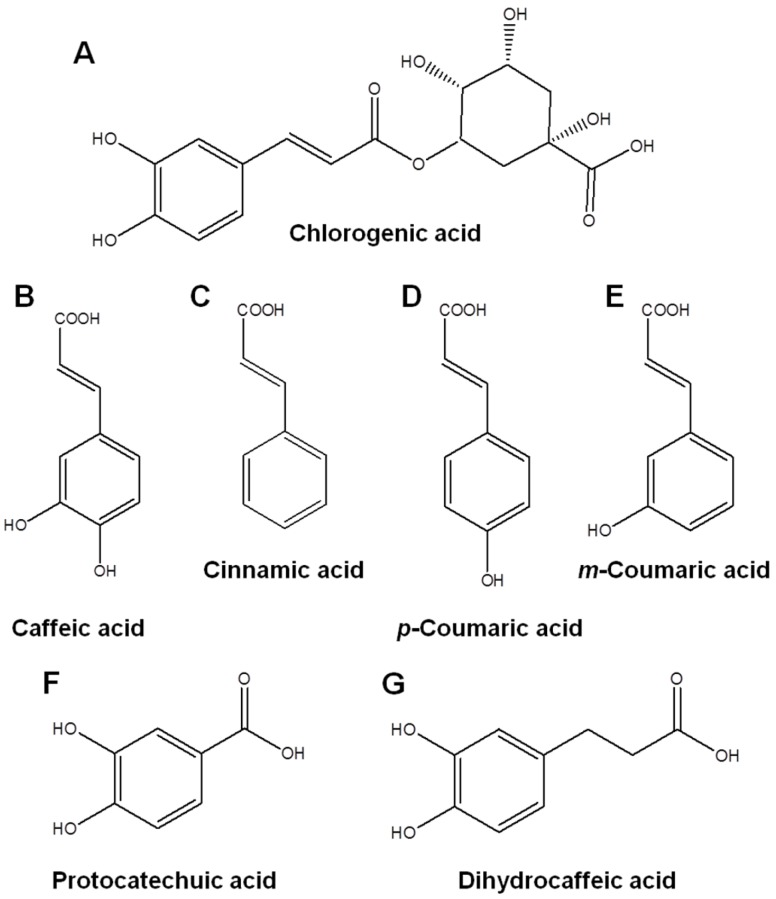
Chemical structures of analogues derived from chlorogenic acid (CHA) and caffeic acid (CA). Chemical structure of CHA (**A**); CA (**B**); cinnamic acid (**C**); *p*-coumaric acid (**D**); *m*-coumaric acid (**E**); protocatechuic acid (**F**); and dihydrocaffeic acid (**G**).

Therefore, we investigated the inhibitory mechanisms of CHA and CA against oxidative stress-induced IL-8 production in human intestinal epithelial Caco-2 cells. Furthermore, we elucidated the functional moieties mediating these anti-inflammatory effects using structural analogs (Figure 1) of CHA and CA.

## 2. Materials and Methods

### 2.1. Reagents

The Caco-2 cell lines were obtained from the American Type Culture Collection (Rockville, MD, USA). Dulbecco’s modified Eagle’s medium (DMEM) was purchased from Wako Pure Chemicals (Osaka, Japan). Fetal bovine serum (FBS), penicillin-streptomycin and non-essential amino acids (NEAA) were purchased from Gibco (Gaithersburg, MD, USA). CHA (PubChem CID: 1794427), CA (PubChem CID: 689043), *p*-coumaric acid (PubChem CID: 637542), and cinnamic acid (PubChem CID: 444539) were purchased from Sigma (St. Louis, MO, USA). *m*-coumaric acid (PubChem CID: 637541), protocatechuic acid (PubChem CID: 72), and dihydrocaffeic acid (PubChem CID: 348154) were purchased from Wako (Osaka, Japan). The dual-luciferase reporter assay system was obtained from Promega (Madison, WI, USA). The anti-human nuclear factor kappa-light-chain-enhancer of activated B cells (NFκB) p65 and IκB kinase (IKK) α/β (p-IKKα/β) antibodies were purchased from Santa Cruz Biotechnology (Santa Cruz, CA, USA). Protein kinase D (PKD) and p-PKD antibodies were purchased from Cell Signaling Technology (Beverly, MA, USA), and horseradish peroxidase-linked goat anti-rabbit IgG was from ICN Biomedicals (Aurora, OH, USA). ECL Plus Western blotting detection reagents were obtained from GE Healthcare Amersham Bio-Sciences (Buckinghamshire, UK). 2′,7′-dichlorodihydrofluorescein diacetate (H_2_DCFDA) was purchased from Invitrogen (Carlsbad, CA, USA).

### 2.2. Cell Culture

Caco-2 cells were cultured at 37 °C in humidified air containing 5% CO_2_. The cells were maintained in a 100-mm dish with DMEM containing 1000 mg/L of glucose and supplemented with 10% FBS, 1% NEAA, 200 U/mL of penicillin and 200 μg/mL of streptomycin. The cells were subcultured weekly by trypsinization (0.1% trypsin, 0.5-mM EDTA) and seeded at a density of 2 × 10^5^ cells/well on a 24-well plate. The cells were allowed to grow for 2 weeks for the experiments, and the medium was changed every 2–3 days. The Caco-2 cells were used between passages 40 and 60.

### 2.3. Transient Transfection and Luciferase Assay

The *IL-8* reporter vector was constructed by inserting the human *IL-8* promoter region containing the glucocorticoid-responsive element, transcriptional factor activator protein 1 (*AP-1*), CCAAT/enhancer-binding protein (*C/EBP*), and *NF-κB* consensus element into the *pGL3*-basic vector (Promega, Madison, WI, USA). The *NF-κB* reporter vector was constructed by inserting four *NF-κB* consensus elements (TGGAATTTCC) into the *pGL3*-promoter vector (Promega, Madison, WI, USA).

Caco-2 cells were seeded at 1 × 10^5^ cells/well on 12-well tissue culture plates 1 day prior to transient transfection. At 70% confluency, cells were co-transfected with *pRL-CMV* (the internal control plasmid) and the *pGL3-IL-8* or *NF-κB* vector by using the LipofectAmine Plus reagent (Invitrogen, Carlsbad, CA, USA) according to the manufacturer’s protocol. The transfected cells were allowed to recover overnight at 37 °C and subsequently exposed to 1 mmol/L H_2_O_2_ for 24 h. At the same time, we treated the cells with CHA or CA. The luciferase activity was measured by a Luminometer using the dual-luciferase reporter assay system (Promega, Madison, WI, USA) according to the manufacturer’s instructions. 

### 2.4. Western Blot Analysis

In order to analyze the nuclear translocation of p65, the cells were suspended in buffer A (10 mmol/L HEPES, 10 mmol/L KCl, 0.1 mmol/L EDTA, 0.1 mmol/L EGTA, 10% NP-40, 1 mmol/L DTT, 0.5 mmol/L PMSF, and 1 μg/mL of a protease inhibitor cocktail from Sigma, at pH 7.9). The cells were vigorously vortexed and centrifuged. After removing the supernatant, the cell pellet was suspended in buffer C (20 mmol/L HEPES, 25% *v/v* glycerol, 0.4 M NaCl, 1 mmol/L EDTA, 1 mmol/L EGTA, 1 mmol/L DTT, 1 mmol/L PMSF, and 1 μL/mL of a protease inhibitor cocktail from Sigma, at pH 7.9). After vortexing and centrifuging, the supernatant was used as the nuclear extract. Using 20 μg of nuclear proteins, the nuclear translocation of NF-κB p65 was detected by electrophoresis and antibody response. The analysis was carried out with a chemiluminescent substrate (ECL; Amersham Biosciences).

To analyze the phosphorylated and total protein level of IKK and PKD, the cells were suspended in lysis buffer (10 mmol/L Tris-HCl, 150 mmol/L NaCl, 1% Nonidet P-40, 1 mmol/L EDTA, 20 mmol/L NaF, 100 μmol/L sodium orthovanadate, 20 μmol/L PMSF, and 100 μg/mL of a calpain inhibitor, at pH 7.8). The cells were vigorously vortexed and centrifuged, and the supernatant was used as the whole cell extract. Using 20 μg of cytosolic proteins, the phosphorylation of IKK and PKD were detected by electrophoresis and antibody response as above.

### 2.5. Measurement of Intracellular ROS Generation

The intracellular ROS level was detected by using H_2_DCFDA. H_2_DCFDA is a cell-permeable indicator for ROS that is non-fluorescent until the acetate groups are removed by intracellular esterases, which allows oxidation to occur within the cell, causing irreversible conversion to the fluorescent form, DCF. For ROS measurements, Caco-2 cells were incubated for 5 min with HBSS containing 10 μmol/L H_2_DCFDA after pre-treatment with CHA or CA. The cells were washed with HBSS for removal of extracellular H_2_DCFDA, and then treated with 2 mmol/L H_2_O_2_ for 1 h. The fluorescence was measured by a plate reader with an excitation wavelength of 485 nm and an emission wavelength of 538 nm.

### 2.6. ROS-Scavenging Activity

The scavenging activities were measured by monitoring the capacity to reduce superoxide, hydrogen peroxide, and hydroxyl radical generation. These ROS were detected by using 2-methyl-6-*p*-methoxyphenylethinylimidazopyrazinone (MPEC; ATTO, Tokyo, Japan) and xylenol orange (Sigma, and a radical catch kit (ALOKA, Tokyo, Japan). The scavenging activity (%) was determined using the following formula:

Scavenging activity = [(activity of control) − (activity of sample)/(activity of control)] × 100(1)

### 2.7. Enzyme-Linked Immunosorbent Assay

Confluent monolayers of Caco-2 cells on the 24-well plate were exposed to a culture medium containing 2 mmol/L H_2_O_2_ and CA derivatives. After incubation, the level of IL-8 in the cell supernatant was determined using commercially available enzyme-linked immunosorbent assay kits (Pierce, Rockford, IL, USA) according to the manufacturer’s instructions. 

### 2.8. RNA Isolation and Real-Time PCR

Total RNA was isolated from the cells by using Isogen (Nippon Gene, Tokyo, Japan) according to the manufacturer’s instructions. Reverse transcription of the RNA was performed by using an ExScript RT reagent kit (Takara, Otsu, Japan). First-strand cDNA was prepared from 1 μg of the total RNA. 

The real-time PCR reaction was performed in a 10 μL volume containing 1 μg of cDNA, 0.5 μmol/L of each primer, and 2 × SYBR Premix Ex Taq™. Each sample was denatured at 95 °C for 15 min and subsequently subjected to 40 cycles of 15 s at 95 °C for denaturation, 15 s at the appropriate annealing temperature (56–57 °C), and 10 s at 72 °C for elongation. The primer sequences were as follows: *human IL-8*, 5′-AGAGTGATTGAGAGTGGACC-3′ (forward) and 5′-ACTTCTCCACAACCCTCTG-3′ (reverse); and *β-actin*, 5′-CCACGAAACTACCTTCAAC-3′ (forward) and 5′-GATCTTCATTGTGCTGGG-3′ (reverse). The real-time PCR reactions were run on a Lightcycler (Roche Applied Science, Penzberg, Germany). The gene expression levels were normalized to the level of the housekeeping gene (β-actin). Relative gene expression changes are reported as number-fold changes compared to the control samples.

### 2.9. Statistical Analysis

Each result is expressed as the mean ± the standard error (SE). The data were tested with Tukey’s multiple-range test when significant differences (*p* < 0.05) were obtained by one-way ANOVA.

## 3. Results

### 3.1. Inhibitory Effect of CHA and CA on H_2_O_2_-Induced IL-8 and NF-κB-Dependent Transcriptional Activities in Caco-2 Cells

**Figure 2:**
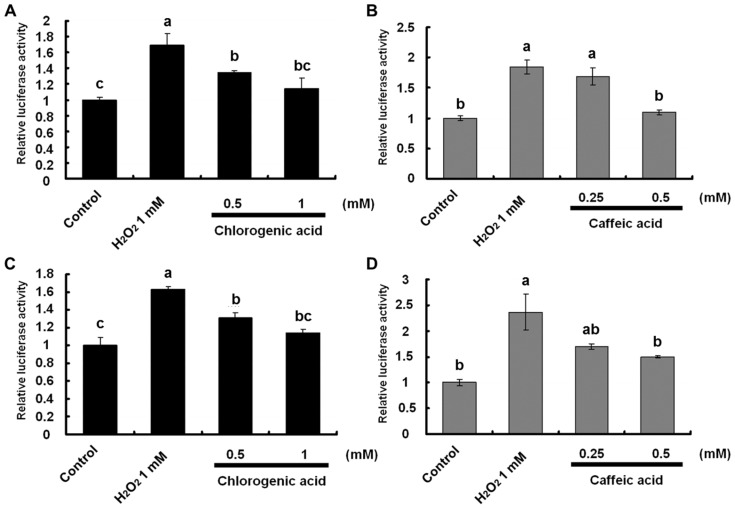
Inhibitory effect of chlorogenic acid (CHA) and caffeic acid (CA) on hydrogen peroxide (H_2_O_2_)-induced interleukin (IL)-8 and nuclear factor kappa-light-chain-enhancer of activated B cells (NF-κB)-dependent transcriptional activities in Caco-2 cells. (**A**,**B**) Undifferentiated Caco-2 cells were co-transfected with the pGL-3 basic vector containing *IL-8* promoter region (*C/EBP*, *AP-1*, and *NF-κB*) and *pRL-CMV* control vector; (**C**,**D**) Undifferentiated Caco-2 cells were co-transfected with the pGL-3 promoter vector containing four binding sites (5′-TGGAATTTCCTCT-3′) of *NF-κB* and *pRL-CMV* control vector; (**A**,**C**) Cells were also treated with 1 mM H_2_O_2_ and 0.5–1 mM CHA; and (**B**,**D**) 0.25–0.5-mM CA for 24 h. *IL-8* and *NF-κB* transcriptional activities were estimated using a luciferase assay. Values are mean ± SE (*n* = 3). Means that have no letter in common are significantly different from each other (*p* < 0.05) by Tukey’s test.

To investigate *IL-8* transcriptional activity, Caco-2 cells co-transfected with *pRL-CMV* and *pGL3-IL-8* were treated with 1 mmol/L H_2_O_2_ and CHA for 24 h. Treatment with CHA and CA inhibited the H_2_O_2_-induced *IL-8* transcriptional activity dose-dependently (Figure 2A,B). These results suggest that the inhibitory effects of CHA and CA on H_2_O_2_-induced *IL-8* transcriptional activity were associated with suppression of transcriptional factors such as *AP-1*, *C/EBP*, and *NF-κB*.

Among these factors, we particularly focused on NF-κB activation because it is crucial to immune modulation, inflammation, and cell proliferation responses [13]. Furthermore, it was reported that the activation of NF-κB plays a key role in IL-8 production induced by oxidative stress (H_2_O_2_) or pro-inflammatory cytokines such as tumor necrosis factor (TNF)-α in intestinal epithelial Caco-2 cells [14]. Therefore, we investigated the effects of CHA and CA on the activation of NF-κB-dependent transcription induced by H_2_O_2_ using the *pNF-κB* *x 4-luc* reporter vector. The results showed that the *NF-κB* transcriptional activity increased compared with that of the control in response to treatment with H_2_O_2_. In addition, CHA and CA significantly suppressed *NF-κB*-dependent transcriptional activity in human intestinal Caco-2 cells (Figure 2C,D).

### 3.2. CHA and CA Suppressed Activation of H_2_O_2-_Induced PKD-IKK-NF-κB Signaling

**Figure 3:**
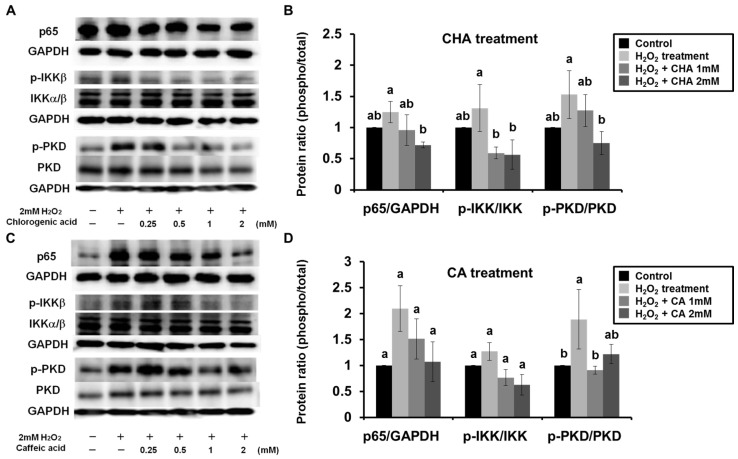
Chlorogenic acid (CHA) and caffeic acid (CA) suppressed activation of hydrogen peroxide (H_2_O_2_)-induced protein kinase D (PKD)-I-κB kinase (IKK)-nuclear factor kappa-light-chain-enhancer of activated B cells (NF-κB) signaling. Caco-2 cells were pre-incubated for 3 h with 0.25–2 mM CHA or CA, and then exposed to 2 mM H_2_O_2_ for 1 h (p65), 10 min (IKK), or 5 min (PKD). (**A**) CHA and (**C**) CA were also added to Caco-2 cells at a concentration equal to that used for pretreatment. For quantification of p65, whole proteins extracted from Caco-2 cells were separated into cytosol and nuclear fractions. In addition, whole proteins extracted from Caco-2 cells were investigated for activation of IKK or PKD by comparing regular and phospho-IKK or phospho-PKD using Western blotting with GAPDH as a housekeeping protein. Data represent three independent experiments, and densitographic analysis of these bands was performed (**B**,**D**). Values are mean ± SE (*n* = 3). Means that have no letter in common are significantly different from each other (*p* < 0.05) by Tukey’s test.

The activation of NF-κB is generally accompanied by nuclear translocation of the p65 subunit induced by degradation of the IκB subunit. Therefore, we examined the effects of CHA on the nuclear translocation of NF-κB (p65) in H_2_O_2_-treated Caco-2 cells. The results revealed that the H_2_O_2_-induced nuclear translocation of p65 was dose-dependently inhibited by treatment with CHA (Figure 3A,B). Furthermore, we investigated the activation of the IKK as an upstream molecule of NF-κB by measuring its degree of phosphorylation. Figure 3A,B showed that CHA suppressed the H_2_O_2_-induced phosphorylation of IKK in Caco-2 cells. Next, we investigated signaling upstream of IKK activation. It is known that IKK is activated by various stimuli such as oxidative stress, TNF-α, IL-1β, and toll-like receptor ligands, which have different signaling pathways. These pathways involve specific signaling molecules such as interleukin-1 receptor-associated kinase, mitogen-activated protein kinase, and NF-κB-inducing kinase [13,15] for phosphorylation of IKK. Among these molecules, PKD is specifically activated by oxidative stress. Therefore, we examined the effect of CHA on the phosphorylation of PKD induced by H_2_O_2_ in Caco-2 cells. The result showed that the phosphorylation of PKD was strongly induced following treatment with H_2_O_2_, and CHA suppressed this activation of PKD (Figure 3A,B). We also investigated the effect of CA on the PKD-IKK-NF-κB signaling pathway. Treatment with CA inhibited activation of the PKD-IKK-NF-κB signaling pathway in Caco-2 cells (Figure 3C,D). These results demonstrate that both CHA and CA inhibited the H_2_O_2_-induced IL-8 production via suppression of the PKD-IKK-NF-κB signaling pathway in Caco-2 cells.

### 3.3. ROS Scavenging ROS Capacity of CHA and CA 

**Figure 4:**
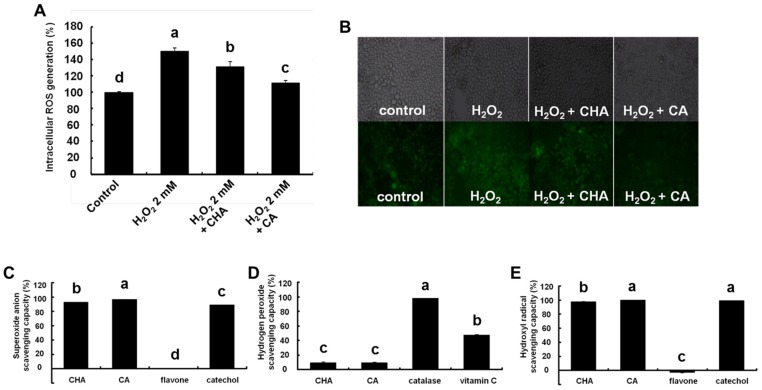
Reactive oxygen species (ROS) scavenging capacity of chlorogenic acid (CHA) and caffeic acid (CA). Caco-2 cells were pre-incubated for 3 h with 1 mM CHA or CA and then 10 μM H_2_DCFDA was added for 5 min after washing twice with HBSS. Caco-2 cells were exposed to 2 mM hydrogen peroxide (H_2_O_2_) for 1 h after washing twice with HBSS. (**A**) H_2_DCFDA fluorescence was measured using a plate reader with an excitation and emission wavelengths of 485 and 544 nm, respectively; (**B**) Caco-2 cells cultured on an eight-chambered cover glass were treated under similar conditions, and fluorescence was observed by fluorescence microscopy. Three ROS were observed in cells including (**C**) superoxide; (**D**) H_2_O_2_; and (**E**) hydroxyl radical. Superoxide produced by xanthine oxidase was detected using a luminometer. H_2_O_2_ and hydroxyl radicals induced by Fenton reaction were measured using a microplate reader and luminometer. Catechol, catalase, and vitamin C were used as positive controls, and flavone was a negative control. Values are mean ± SE (*n* = 3). Means that have no letter in common are significantly different from each other (*p* < 0.05) by Tukey’s test.

Our results showed that CHA and CA suppressed the PKD-IKK-NF-κB signaling pathway activated by oxidative stress. Therefore, we investigated the possibility that CHA and CA could scavenge intracellular ROS generated by H_2_O_2_ using H_2_DCFDA [16]. The results revealed that CHA and CA significantly inhibited H_2_O_2_-stimulated intracellular ROS generation (Figure 4A). Moreover, as shown in Figure 4B, CHA and CA effectively reduced the H_2_O_2_-induced intracellular ROS observed using fluorescence microscopy. These results demonstrate that the inhibitory effect of CHA and CA on H_2_O_2_-induced IL-8 production was mediated by their ROS scavenging capacity.

To identify the type of intracellular ROS involved, we investigated the scavenging activities of CHA and CA using superoxide, H_2_O_2_, and hydroxyl radicals in vitro. CHA and CA significantly scavenged both superoxide produced by xanthine oxidase and hydroxyl radicals produced by the fenton reaction, but they had no effect on H_2_O_2_ peroxide (Figures 4C–E). Therefore, we propose that the inhibitory effects of CHA and CA on IL-8 production via activation of PKD-IKK-NF-κB signaling were due to the suppression of intracellular ROS generation, especially superoxide, and hydroxyl radical production. 

### 3.4. Effects of CHA and CA Derivatives on H_2_O_2_-Induced IL-8 Production

**Figure 5:**
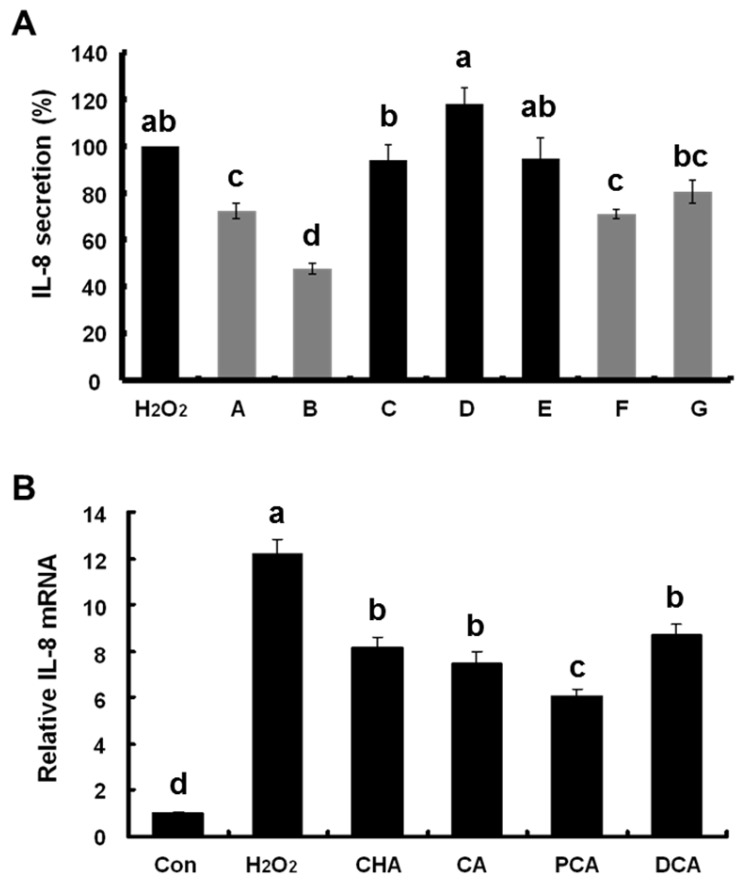
Effects of chlorogenic acid (CHA) and caffeic acid (CA) derivatives ((**A**–**G**), as shown in Figure 1) on hydrogen peroxide (H_2_O_2_)-induced interleukin (IL)-8 production. (**A**) Caco-2 cells were exposed to 2 mM H_2_O_2_ and treated with 1 mM of each compound. After 24 h, culture medium was collected and IL-8 secretion was determined using enzyme-linked immunosorbent assay (ELISA); (**B**) IL-8 mRNA expression was measured 3 h after treatment using real-time reverse transcription polymerase chain reaction (qRT-PCR). Values are mean ± SE (*n* = 3). Means that have no letter in common are significantly different from each other (*p* < 0.05) by Tukey’s test.

To reveal the functional moieties responsible for the anti-inflammatory effects of CHA and CA, we examined the effect of various CHA and CA derivatives such as cinnamic acid, *p*-coumaric acid, *m*-coumaric acid, protocatechuic acid (PCA), and dihydrocaffeic acid (DCA, Figure 1) on H_2_O_2_-induced IL-8 secretion in Caco-2 cells. As shown in Figure 5A, CHA, CA, PCA, and DCA having two hydroxyl groups (catechol group) significantly inhibited H_2_O_2_-induced IL-8 secretion while cinnamic acid (no hydroxyl group), *p*-coumaric acid (*para*-hydroxyl group), and *m*-coumaric acid (*meta*-hydroxyl group) did not. This result indicates that CHA, CA, PCA, and DCA, which inhibited IL-8 production, all commonly possessed two hydroxyl groups (catechol group) in their structure. 

To confirm the anti-inflammatory effects of these groups, we investigated whether CHA, CA, PCA, and DCA inhibited expression of *IL-8* mRNA induced by H_2_O_2_. CHA, CA, PCA, and DCA significantly suppressed H_2_O_2_-induced *IL-8* mRNA expression in Caco-2 cells (Figure 5B). The suppressive effect of the catechol group on H_2_O_2_-induced IL-8 production in Caco-2 cells was not observed because the catechol (intact form) caused damage to the cells indicating its cytotoxic nature [17]. However, our findings strongly suggest that the catechol group in CHA and CA plays a crucial role in the inhibition of IL-8 production induced by oxidative stress.

## 4. Discussion

In the present study, the Caco-2 cells were exposed to 1–2 mmol/L of H_2_O_2_ as an oxidative stressor. However, the concentration of H_2_O_2_ that may cause cell damage or death by necrosis or apoptosis is above a certain concentration range. Therefore, we observed the morphology of the cells using microscopy to determine the optimal concentration of H_2_O_2_ that induced cell damage or death. The result showed there was no significant change in cell morphology at the concentration range of 0–2 mmol/L. We also evaluated cytotoxicity by measuring lactate dehydrogenase (LDH) levels in the cells treated with H_2_O_2_ (2 mmol/L) and CHA or CA (0–4 mmol/L). The LDH levels of the cells treated with these compounds were not significantly different from the control [18]. These results indicate that the human intestinal epithelial Caco-2 cells were not damaged by these treatments.

We found that both CHA and CA significantly inhibited IL-8 production induced by H_2_O_2_ in human intestinal Caco-2 cells. CA exhibited a stronger anti-inflammatory activity than CHA against the IL-8 production. Furthermore, treatment with CA at the same concentration (0.5 mmol/L) showed a higher inhibition of the *IL-8* transcriptional activity than treatment with CHA did (59.3% and 79.8%, respectively). To explain this discrepancy, we hypothesized that CHA and CA may be absorbed via different absorption mechanisms. It has been reported that CHA is absorbed mainly via transcellular diffusion while CA is absorbed via both transcellular diffusion and the monocarboxylic acid transporter (MCT) in Caco-2 cells [19]. Furthermore, the amount of CA absorbed is three times more than that of CHA in humans although both compounds are absorbed in the small intestine [20]. Therefore, we propose that the MCT-mediated uptake of CA may explain our experimental results, which showed CA to have a stronger effect than CHA did at the same dose range. 

Generally, a cup of brewed commercial coffee (200 ml) contains 96 mg of CHA (1.4 mM), and a habitual coffee drinker may consume 500–1000 mg of CHA daily [21,22]. By ileostomy human study, about 33% of the ingested CHA was absorbed to intact form in the foregut, and the rest (about 67% of the ingested CHA) reached the hindgut and was exposed to intestinal epithelial cells [20]. In other words, uptake of a cup of coffee is able to make the reaching of CHA to 0.938 mM (approximately 1 mM) of dosages in intestinal epithelium. Therefore, we suggest that intake of two cups of coffee may help to attenuate intestinal inflammation induced by oxidative stress.

The activation of the PKD-IKK-NF-κB signaling pathway by oxidative stress was previously reported by Storz et al. [23]. Briefly, oxidative stress induces PKD activation through phosphorylation of PKD by both Src-Abl-mediated tyrosine phosphorylation (Y463) and PKC-mu-mediated activation-loop phosphorylation (S744/748). Activated PKD subsequently activates NF-κB via the IKK complex, especially IKK-β. Then, the IκB proteins are rapidly phosphorylated and degraded by the proteasome, and the freed NF-κB p65/p50 heterodimer [24] translocates into the nucleus to regulate the expression of multiple target genes [23,25,26]. However, it is important that the signals activated by oxidative stress vary in different cell types [27]. In this study, we revealed that the human intestinal epithelial Caco-2 cells were induced the activation of the PKD-IKK-NF-κB signaling pathway by oxidative stress. Furthermore, we found that the activation of PKD-IKK-NF-κB signaling pathway induces production of the pro-inflammatory cytokine IL-8, resulting in activation of a PKD-IKK-NF-κB signaling pathway in Caco-2 cells.

In the present study, we revealed that the CHA and CA effectively inhibited a PKD-IKK-NF-κB-IL-8 signaling pathway by scavenging intracellular ROS. However, this mechanism of CHA and CA might be associated to other signaling transduction such as antioxidant and redox signaling pathways. Representatively, HO-1 (heme oxygenase 1) and NQO-1 (NAD(P)H dehydrogenase 1) as phase 2 antioxidant enzymes have been known to act to protect against oxidative stress [28]. Induction of the enzymes is able to suppress oxidative damages and pro-inflammatory signals caused by oxidative stress. Furthermore, it was reported that CHA upregulated the HO-1 and NQO-1 by activating Nrf2 (Nuclear factor erythroid 2-related factor 2) and phosphorylating Akt, also known as protein kinase B [29]. Therefore, in the present study, CHA and CA might inhibit PKD-IKK-NF-κB-IL-8 signaling pathway through induction of Akt-Nrf2-HO-1 signals. However, it has not yet been known which CHA preferentially act either NF-κB-related signals or Nrf2-related signals in the cell modification caused by oxidative stress. Underlying mechanisms of CHA and CA will be clarified in further studies.

In this study, we found that the anti-inflammatory effects of CHA and CA depended on the presence of the catechol group in their chemical structures. PCA and DCA, which contain a catechol group, showed suppressive effects against IL-8 production induced by H_2_O_2_. It was reported that hydroxyl groups on phenolic acids acted as effective moieties for their antioxidant activities, which depended on the number of hydroxyl groups as follows: mono-hydroxy < di-hydroxy (catechol) < tri-hydroxyphenolic acids [30]. In this study, cinnamic acid (no hydroxyl group) as well as *p*- and *m*-coumaric acids (a hydroxyl group) did not inhibit H_2_O_2_-induced IL-8 secretion. In contrast, CHA, CA, PCA, and DCA, which contain di-hydroxyl (catechol) groups, suppressed IL-8 production. These results indicate that the anti-inflammatory activity of phenolic acids did not completely correlate with their antioxidant activity. Therefore, we suggest that phenolic acids containing catechol groups exert anti-inflammatory effects; however, compounds without catechol or containing single hydroxyl groups do not or their antioxidant activities are not strong enough to induce anti-inflammation.

## 5. Conclusions

**Figure 6:**
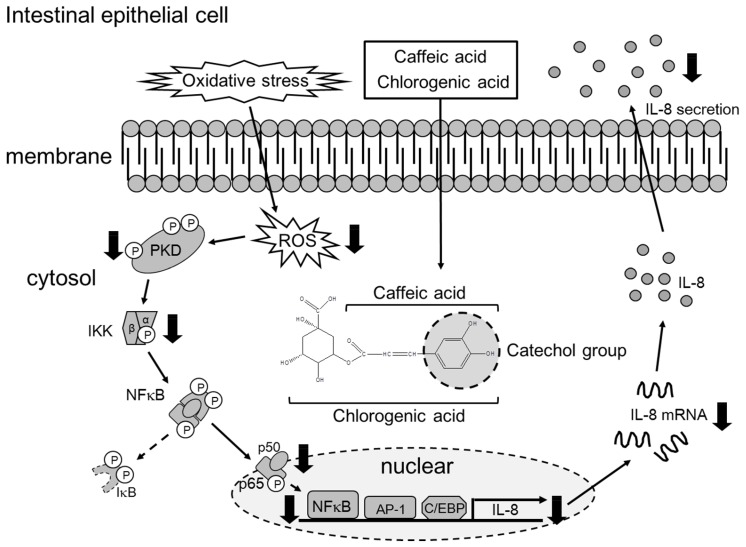
Proposed anti-inflammatory mechanism of chlorogenic acid (CHA) and caffeic acid (CA) in human intestinal epithelial cells.

In the present study, we elucidated the anti-inflammatory mechanism of CHA and CA in human intestinal epithelial cells (Figure 6). CHA and CA inhibited activation of the PKD-IKK-NF-κB-IL-8 signaling pathway by scavenging intracellular ROS generated by oxidative stress. This study also demonstrated that the anti-inflammatory effects of CHA and CA were dependent on their catechol groups. Therefore, we believe that CHA, CA, and other compounds possessing the catechol group might be useful in contributing to preventing intestinal inflammatory diseases.
